# Supplementary material for: The role of andrographolide as a potential anticancer agent against gastric cancer cell lines: a systematic review
Source: PeerJ. 2024 Nov 20;12:e18513. doi: 10.7717/peerj.18513 (PMC11585289; doi:10.7717/peerj.18513)
Supplement: Supplemental Information 3 [file peerj-12-18513-s003.docx]

**Title**

The Role of *Andrographis paniculata* as a Natural Product with Potential Gastric Cancer Treatment Properties: A Systematic Review

**Keywords**

“*Andrographis paniculata*” OR andrographolide” AND “gastric cancer” OR “gastric carcinoma” OR “stomach cancer" OR “stomach carcinoma”

**Search strategy**

| **No** | **Databases** | **Search strategy** | **Total (*N*)** |
| --- | --- | --- | --- |
| 1 | **PubMed** | ((andrographis paniculata[Title/Abstract]) OR (andrographolide[Title/Abstract])) AND ((((gastric cancer[Title/Abstract]) OR (gastric carcinoma[Title/Abstract])) OR (stomach cancer[Title/Abstract])) OR (stomach carcinoma[Title/Abstract])) | **15** |
| 2 | **Scopus** | TITLE-ABS ( andrographis AND paniculata ) OR TITLE-ABS ( andrographolide ) AND TITLE-ABS ( gastric AND cancer ) OR TITLE-ABS ( gastric AND carcinoma ) OR TITLE-ABS ( stomach AND cancer ) OR TITLE-ABS ( stomach AND carcinoma ) | **22** |
| 3 | **ScienceDirect** | (andrographis paniculata) (andrographolide) (gastric cancer) (gastric carcinoma) (stomach cancer) (stomach carcinoma) | **39** |
| 4 | **Google Scholar** | allintitle: ("andrographis paniculata" OR andrographolide) AND ("gastric cancer" OR "gastric carcinoma" OR "stomach cancer" OR "stomach carcinoma") | **10** |
| 5 | **Web of Science** | (TI=("andrographis paniculata" OR "andrographolide")) AND TI=(("gastric cancer" OR "gastric carcinoma" OR "stomach cancer" OR "stomach carcinoma")) | **7** |

Total: 93 references

Duplicates: 19 references

Start screening with: 74 references
