# Supplementary material for: The role of andrographolide as a potential anticancer agent against gastric cancer cell lines: a systematic review
Source: PeerJ. 2024 Nov 20;12:e18513. doi: 10.7717/peerj.18513 (PMC11585289; doi:10.7717/peerj.18513)
Supplement: Supplemental Information 2 [file peerj-12-18513-s002.pdf]

Dr. Tuan Noorkorina Tuan Kub
Department of Medical Microbiology & Parasitology
School of Medical Sciences
Universiti Sains Malaysia
16150 Kubang Kerian
Kelantan.

Dear Editor-in-Chief,

I wish to submit a review article entitled “The role of andrographolide as a potential anticancer agent against gastric cancer cell lines: a systematic review” for consideration of publication in *Peer J*.
We believe that our research will appeal to the readership of this journal, particularly for those with special interest in cell biology, immunology, microbiology and pharmacology.
To best of our knowledge and based on our search, there is still lack of systematic review conducted on andrographolide applications onto gastric cancer cells. The reviews were only focused on andrographolide applications onto other cancer cells.

The rationale of study is that the gastric cancer is the third leading causes of cancer-related deaths worldwide. equating to 1 in every 12 deaths globally. Despite advances in treatment, the prognosis for gastric cancer remains poor, particularly in advanced stages. There is a pressing need for new therapeutic strategies that can improve patient outcomes. Current treatments for gastric cancer, including surgery, chemotherapy, and radiotherapy, often have limited effectiveness and significant side effects. Resistance to chemotherapy is a major challenge, necessitating the search for novel agents that can overcome these limitations. Natural compounds have historically been a rich source of therapeutic agents. Many studies using natural compound such as andrographolide has been done as they often possess unique mechanisms of action and can offer fewer side effects compared to conventional chemotherapy drugs. Exploring natural compounds for anticancer properties is a promising area of research. Andrographolide is a diterpenoid lactone derived from the plant *Andrographis paniculata*, traditionally used in Asian medicine. It has demonstrated various pharmacological activities, including anti-inflammatory, antiviral, and anticancer properties. Previous studies have shown that andrographolide can induce apoptosis, inhibit proliferation, and affect various signalling pathways involved in cancer progression. Understanding these mechanisms in the context of

gastric cancer can provide insights into its potential efficacy and therapeutic applications. Many existing systematic reviews focus on conventional chemotherapeutic agents, targeted therapies, and immunotherapies. There is often a lack of comprehensive reviews on natural compounds, such as andrographolide, which have shown promise in *in vitro* studies. Therefore, there is an urgent need for comprehensive overview of existing research. This systematic review can lay the groundwork for future clinical trials. By compiling and analysing existing preclinical data, researchers can better design studies to evaluate the safety, efficacy, and dosage of andrographolide in human patients into the anticancer treatment of gastric cancer.

Thank you for your consideration of this manuscript. We appreciate your time and look forward to your response.

Best regards,

Dr. Tuan Noorkorina Tuan Kub
